# Job demands-resources on digital gig platforms and counterproductive work behavior

**DOI:** 10.3389/fpsyg.2024.1378247

**Published:** 2024-09-17

**Authors:** Shanshan Zhao, Yanfeng Liu

**Affiliations:** ^1^School of Business and Management, Jiaxing Nanhu University, Jiaxing, China; ^2^School of Management of Technology, Pukyong National University, Busan, Republic of Korea; ^3^Department of International Trade and Logistics, Chung-Ang University, Seoul, Republic of Korea

**Keywords:** gig economy, digital gig platform, counterproductive work behavior, job demands-resources model, job engagement theory, second-order structural equation model

## Abstract

**Introduction:**

With the rapid expansion of digital gig platforms, counterproductive work behavior among gig workers has become increasingly prominent, adversely impacting the platform’s reputation, operational efficiency, and user experience. This study aims to explore how job demands and job resources influence counterproductive work behavior among gig workers.

**Methods:**

Grounded in the Job Demands-Resources model and Job Engagement Theory, this study develops a second-order chain mediation structural model to analyze the effects of job demands (Work pace/workload, physical demands, psychological demands, and customer-related social stressors) and job resources (Compensation, job security, learning opportunities, and opportunities for professional development) on counterproductive work behavior. Cognitive, emotional, and behavioral engagement are also examined as mediators.

**Results:**

Job demands positively influence counterproductive work behavior, while job resources have a negative impact. Cognitive, emotional, and behavioral engagement each negatively affect counterproductive work behavior. Additionally, platform formalization moderates the negative influence of gig workers’ engagement on counterproductive work behavior.

**Discussion:**

This research provides a comprehensive theoretical framework for digital gig platform managers to understand and predict gig workers’ counterproductive work behavior. It also offers practical implications for optimizing the work environment, enhancing job engagement, and mitigating counterproductive work behavior, thus fostering mutual development between gig workers and the platform.

## 1 Introduction

With the rapid rise of the digital economy, digital gig platforms have emerged as a new form of employment, attracting a large number of workers (Wan et al., 2024). By the end of 2021, the number of gig workers in China had reached approximately 200 million (National Bureau of Statistics of China). However, these platforms also face numerous challenges, one of which is Counterproductive work behavior among gig workers (Zhang et al., 2023). Counterproductive work behavior refers to actions by employees that deliberately harm the organization and its stakeholders’ legitimate interests (Sivarajan et al., 2021). On digital gig platforms, this behavior is particularly concerning. Firstly, digital gig platforms typically employ short-term, temporary work relationships rather than traditional formal employment, which complicates the identification and management of counterproductive work behavior (Gandhi and Sucahyo, 2021). Secondly, platforms use algorithms and rules to manage work allocation and performance, but the opacity of algorithms and rigidity of rules may cause gig workers to perceive unfairness and dissatisfaction, increasing the risk of counterproductive work behavior (Zhang et al., 2023). Moreover, although platforms offer significant work autonomy, allowing gig workers to flexibly schedule their work, this autonomy, lacking effective oversight, may also foster counterproductive work behavior (Mohsin et al., 2022). Finally, the lack of social support and security also affects gig workers’ motivation and loyalty, further exacerbating the risk of counterproductive work behavior (Nilsen and Kongsvik, 2023). Thus, as dependence on the gig economy grows and unique challenges arise, effectively managing organizational behavior and reducing counterproductive work behavior among gig workers has become a pressing issue (Zhang et al., 2023).

Currently, although some scholars have explored Counterproductive work behavior among gig workers on digital platforms from perspectives such as psychological contracts, algorithmic management, and negative customer treatment (Xiongtao et al., 2021; Sivarajan et al., 2021; Zhang et al., 2023; Zhao et al., 2007), research on this topic remains in its infancy. Moreover, there is a lack of focus on how the work environment on digital gig platforms influences such behavior. To address this research gap, this study employs the Job demands-resources model (JD-R model) to systematically identify and categorize work environment factors on digital gig platforms and to investigate how these factors influence counterproductive work behavior, providing a comprehensive framework for understanding and predicting counterproductive work behavior among gig workers on digital platforms (Bakker et al., 2007). Specifically, the JD-R model divides the work environment on digital gig platforms into job demands and job resources. Job demands refer to aspects of work that require sustained physical and/or psychological effort or skills from employees (Bakker et al., 2003; Bakker et al., 2007; Demerouti et al., 2001). On digital gig platforms, gig workers often face rapid work pace/workload and heavy workloads, requiring the completion of numerous tasks within short periods (Zhang et al., 2023). Additionally, gig workers such as delivery personnel, movers, or food couriers engage in physically demanding work, which increases physical exhaustion and injury risks (Laskaris et al., 2024). Furthermore, gig workers must manage complex tasks and deal with demanding customers, presenting dual pressures that challenge their psychological state and increase social stress (Xiongtao et al., 2021). Therefore, this study selects work pace/workload, physical demands, psychological demands, and customer-related social stressors as the second-order factors of job demands to accurately reflect the primary work pressures and challenges faced by gig workers on digital platforms. Conversely, job resources are aspects that aid employees in achieving work goals, mitigating job demands and associated costs, and fostering personal growth, learning, and development (Bakker et al., 2003; Bakker et al., 2007; Demerouti et al., 2001). On digital gig platforms, compensation is a critical job resource that impacts gig workers’ economic conditions and quality of life, potentially motivating them to invest more effort in their work. However, inadequate compensation may decrease gig workers’ motivation, especially under high job demands and stress, potentially exacerbating work dissatisfaction and job turnover (Nilsen and Kongsvik, 2023). Additionally, gig workers frequently encounter unstable working conditions and lack social security, such as injury insurance and income stability guarantees, increasing their anxiety and risk awareness (Laskaris et al., 2024). Furthermore, although some platforms offer online training and learning resources to help gig workers enhance their skills and market competitiveness, the quality and availability of these learning opportunities vary across platforms (Zwettler et al., 2023). Lastly, many digital gig platforms primarily offer temporary work, limiting promotion and development opportunities for gig workers, which may decrease their job engagement and increase the risk of counterproductive work behavior (Zwettler et al., 2023). Thus, this study considers compensation, job security, learning opportunities, and opportunities for professional development as the second-order factors of job resources to accurately assess the role of these supportive factors on digital gig platforms.

Furthermore, existing research has shown that job demands and resources can influence counterproductive work behavior through its effect on job engagement (Balducci et al., 2011; Zhao et al., 2007). However, no systematic research has been conducted on how job demands and resources on digital gig platforms affect job engagement and subsequently counterproductive work behavior. In studies of job engagement, Kahn (1990) and Schaufeli et al. (2002) have proposed significant theoretical models (Kahn, 1990; Schaufeli et al., 2002). Schaufeli’s model divides work engagement into dedication, vigor, and absorption, focusing on work task-related enthusiasm but offering limited consideration of overall employee engagement with the work environment and organization (Shuck et al., 2017). In contrast, Kahn’s model, with its division into cognitive engagement, emotional engagement, and behavioral engagement, provides a more comprehensive perspective. Kahn’s model not only assesses engagement in work tasks but also encompasses identification with the work environment, organizational culture, and mission, offering a more accurate reflection of overall engagement levels (Kahn, 1990). Additionally, Kahn’s model exhibits strong explanatory power in understanding counterproductive work behavior, revealing how cognitive biases, emotional states, and behavioral expressions influence counterproductive work behavior (Shuck et al., 2017; Drake, 2012). Thus, this study utilizes Kahn’s Job engagement theory (JE model) to examine how the job demands- resources model affects counterproductive work behavior among gig workers. This multidimensional perspective provides deeper insights into gig workers’ performance across different work environments and offers effective strategies for reducing counterproductive work behavior. Furthermore, in the fields of psychology and management, cognitive and emotional are pivotal factors influencing individual behavior (Dubovi and Tabak, 2021; Yang et al., 2021; Zhao et al., 2007). The behavior of gig workers on digital platforms is not only affected by work task requirements but is also driven by their cognitive and emotional states. Therefore, this study further investigates the impact of cognitive and emotional engagement on behavioral engagement, offering a deeper understanding of the underlying mechanisms of job engagement for gig workers on digital platforms.

Lastly, the governance model of digital gig platforms emphasizes protecting the interests of the platform and its customers, placing gig workers in a relatively disadvantaged position and making it difficult for them to effectively address work-related difficulties (Chen et al., 2023). Additionally, the use of algorithmic management by platforms often results in unclear and opaque operational rules and processes. Therefore, there is an urgent need for appropriate formalization in platform governance to improve gig workers’ perceptions of fairness and reduce the risk of counterproductive work behavior (Chen et al., 2023; Gandhi and Sucahyo, 2021; Rahaman, 2022). This study introduces platform formalization as a moderating variable to explore its role in moderating the relationship between gig workers’ cognitive engagement, emotional engagement, and behavioral engagement, and counterproductive work behavior. The aim is to provide new insights into optimizing digital gig platform governance, promoting the establishment of fair, stable working environments, enhancing gig workers’ work rights, and reducing counterproductive work behavior.

In summary, this study builds a second-order chain mediation structural model based on the JD-R model and JE theory to gain a comprehensive understanding of how the platform work environment affects gig workers’ counterproductive work behavior through individual job engagement. The study aims to address the following questions: (1) What impact do job demands and resources on digital gig platforms have on counterproductive work behavior? (2) How do gig workers’ cognitive engagement, emotional engagement, and behavioral engagement influence counterproductive work behavior, and what is the relationship between these forms of engagement? (3) How do job demands and resources on digital gig platforms influence counterproductive work behavior through gig workers’ cognitive engagement, emotional engagement, and behavioral engagement, and what are the underlying mechanisms? (4) Does platform formalization moderate the relationship between gig workers’ cognitive engagement, emotional engagement, and behavioral engagement, and counterproductive work behavior?

This study holds significant academic and practical implications. Firstly, it innovatively integrates the JD-R model and JE theory to develop a second-order chain mediation structural model, providing a comprehensive theoretical framework for understanding and predicting counterproductive work behavior among gig workers. Additionally, this research offers valuable practical insights for managers of digital gig platforms. By thoroughly examining the relationships among job demands and resources, cognitive engagement, emotional engagement, behavioral engagement, and counterproductive work behavior, managers can more effectively design work environments, enhance job engagement, and mitigate the risk of counterproductive work behavior, thereby informing the development of effective strategies.

The remaining sections of this study are structured as follows: Part Two discusses relevant prior literature on digital gig platforms and gig workers’ counterproductive work behavior, followed by the presentation of the research model and hypotheses. Part Three outlines the research methodology, including questionnaire design and data collection. Part Four reports the data analysis results and discussions of the study. Finally, the theoretical and practical significance of this research are emphasized, along with considerations of its limitations and future prospects.

## 2 Literature review, theoretical model, and research hypotheses

### 2.1 Digital gig platforms and counterproductive work behavior

As an emerging form of employment, digital gig platforms offer gig workers more flexible job opportunities. However, with the widespread adoption of these platforms, the issue of counterproductive work behavior among gig workers has become a focal point for both research and management considerations (Balducci et al., 2011). Unlike traditional employment models where service agencies can curb inappropriate behavior through mandatory regulations, gig workers, operating as freelancers, maintain flexible employment relationships with platforms, lacking mandatory behavioral norms (Xiongtao et al., 2021). Consequently, when faced with platform job requirements and pressures from customers, gig workers may be more prone to exhibiting counterproductive work behavior (Xiongtao et al., 2021). This highlights the unique management challenges within digital gig platforms, necessitating more flexible and innovative management strategies to encourage high-quality service provision. Counterproductive Work Behavior refers to voluntary actions by employees that deliberately harm the organization and its stakeholders (Balducci et al., 2015; Dischner, 2015). Among existing studies, Robinson and Bennett’s (1995) classification method is commonly used. They categorize CWB into two types: Counterproductive Work Behavior directed at the organization (CWB-O) and Counterproductive Work Behavior directed at individuals (CWB-I). Based on the severity of the behavior, Counterproductive Work Behavior is further divided into four categories: production deviance, property deviance, political deviance, and personal aggression (Robinson and Bennett, 1995). On digital gig platforms, production deviance may manifest as workers selectively accepting orders or deliberately rejecting orders during peak times, as well as intentionally delaying work progress or misreporting progress, thus affecting the platform’s productivity (Dischner, 2015). Property deviance involves workers inflating work hours and mileage to increase earnings or engaging in private transactions with customers to bypass platform rules (Gruys and Sackett, 2003). Political deviance is reflected in workers prioritizing merchants or customers with whom they have a personal relationship when accepting orders, discussing customers’ and colleagues’ private matters during work, or even maliciously reporting colleagues to gain more orders and rewards (Behl et al., 2022). Personal aggression includes harassment, verbal abuse, threats, and even stealing customers’ items and food (Xiongtao et al., 2021). Currently, some scholars have explored the counterproductive work behavior of gig workers on digital platforms from perspectives such as algorithmic management, psychological contracts, and negative customer treatment (Xiongtao et al., 2021; Sivarajan et al., 2021; Zhang et al., 2023; Zhao et al., 2007). However, there is a gap in the literature regarding organizational-level factors, specifically the job demands-resources of gig platforms, and their impact on gig workers’ counterproductive work behavior. To address this research gap, this study constructs a second-order chain mediation structural model based on the JD-R model and JE theory. This aims to delve into the relationship and mechanisms between the job demands-resources of digital gig platforms and gig workers’ counterproductive work behavior. Such insights are crucial for the effective management of gig worker behavior on digital gig platforms, aiding platform managers and governments in formulating more flexible and innovative management strategies to enhance the work quality and performance of gig workers.

### 2.2 Theoretical model

The JD-R model is a theoretical framework used to elucidate the impact of the work environment on individuals’ psychological states and behaviors (Balducci et al., 2011; Nilsen and Kongsvik, 2023; Urien Angulo et al., 2019). This framework encompasses physical, psychological, and social/organizational aspects of work, categorized into job demands and job resources. Job demands refer to factors requiring physical or mental effort, while job resources are those that help meet basic job demands, facilitate the achievement of work goals, and stimulate personal growth and development (Bakker et al., 2004; Balducci et al., 2011; Demerouti et al., 2001). The JE theory dissects job engagement into cognitive emotional and behavioral engagement, highlighting the significance of these dimensions on work performance and individual behavior (Dubovi and Tabak, 2021; Huang et al., 2022; Kahn, 1990). Specifically, cognitive engagement pertains to an individual’s understanding and level of commitment to work tasks, such as, “I should work diligently.” Emotional engagement involves the positive and negative emotions experienced by the individual at work, such as, “I am enthusiastic about my work.” Behavioral engagement refers to the individual’s concrete work behaviors, such as, “I put in my best effort to complete my tasks.” These three types of engagement interrelate to form an individual’s overall engagement in their work, significantly impacting their work performance and behavior (Huang et al., 2022; Rich et al., 2010). This study integrates the JD-R Model and JE theory to develop a second-order chain mediation structural model, aiming to explore how job demands and resources on digital gig platforms affect gig workers’ counterproductive work behavior through their cognitive, emotional, and behavioral engagement. This comprehensive theoretical model provides a detailed depiction of the work environment and various dimensions of job engagement on digital gig platforms (Bakker et al., 2007; Kahn, 1990), offering a thorough and systematic framework for researching counterproductive work behavior among gig workers. It aids platform managers in better understanding and predicting gig workers’ counterproductive work behavior.

### 2.3 Research hypotheses

**Figure 1:**
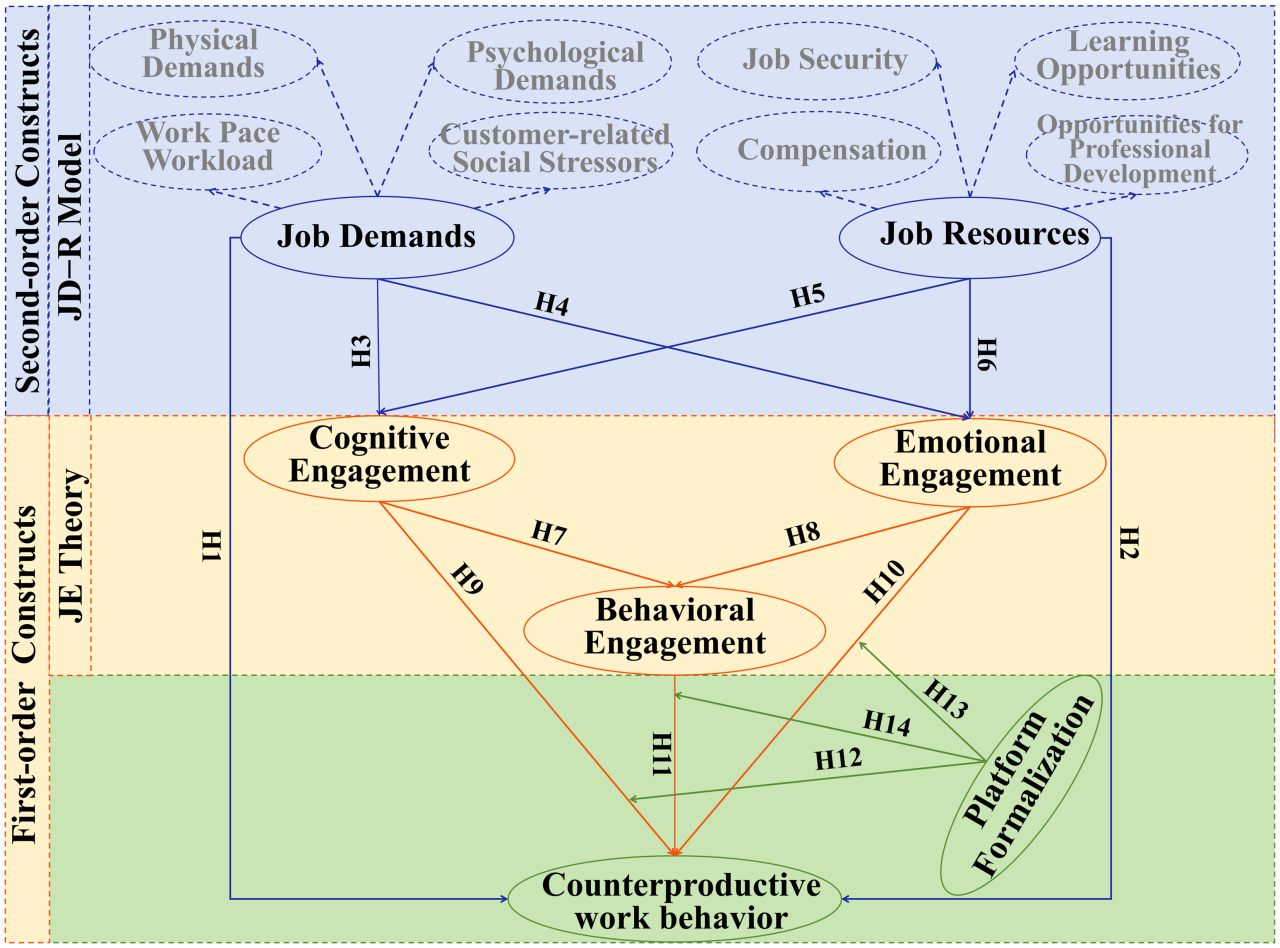
The theoretical model of counterproductive work behavior.

Based on the JD-R model and JE theory, this study constructs a second-order chain mediation structural model and proposes 14 hypotheses (see Figure 1). The model includes first-order constructs, second-order constructs, and a chain mediation structure. Specifically, first-order constructs refer to fundamental constructs directly measured from data; in this study, the work demands and work resources of digital gig platforms serve as second-order constructs. Second-order constructs are higher-level constructs composed of multiple first-order constructs; in this study, they include: work demands comprising work pace/workload, physical demands, psychological demands, and customer-related social stressors; Work resources comprising compensation, job security, learning opportunities, and opportunities for professional development. The chain mediation structure is used to analyze how the independent variables influence the dependent variable through a series of mediators (i.e., mediation chains). In this study, we have constructed a chain mediation model using cognitive engagement, emotional engagement, and behavioral engagement as mediators, connecting the independent variables (work demands and work resources) to the dependent variable (counterproductive work behavior) through a chain relationship, with all variables being latent variables.

#### 2.3.1 Job demands-resources and counterproductive work behavior

Job demands refer to the ongoing physical or mental efforts required in work, associated with physiological and psychological costs (Demerouti et al., 2001). Conversely, job resources are factors that facilitate the achievement of work goals, reduce the costs of job demands, and stimulate personal growth and development (Bakker et al., 2007; Demerouti et al., 2001). Existing research indicates that job demands and resources significantly impact individuals’ counterproductive work behavior (Balducci et al., 2011; Urien Angulo et al., 2019; Zhang et al., 2023). Specifically, Balducci et al. (2011) found that job demands, including workload, role conflict, and interpersonal demands, are positively correlated with counterproductive work behavior. In contrast, job resources such as decision authority, social support, and promotion prospects are positively correlated with job engagement, and subsequently negatively correlated with counterproductive work behavior. Research also suggests that job demands exert a positive influence on counterproductive work behavior through negative emotions. Zhang et al. (2023) noted that algorithmic management on digital gig platforms may lead to irregular work hours, excessive work, and social isolation, with algorithmic opacity causing gig workers to feel unfairly treated and even exhibit destructive misconduct. Urien Angulo et al. (2019) also demonstrated that job demands and resources significantly impact counterproductive work behavior among social work professionals. Based on these findings, the following hypotheses are proposed:

*H1*: Job demands have a significantly positive impact on gig workers’ counterproductive work behavior.

*H2*: Job resources have a significantly negative impact on gig workers’ counterproductive work behavior.

#### 2.3.2 Job demands-resources and job engagement

In studies of job engagement, job demands and resources are recognized as significant factors influencing individual job engagement (Kahn, 1990; Bakker et al., 2007; Trépanier et al., 2014). Job engagement refers to the extent to which organizational members invest their “hands, minds, and hearts” into their work roles, encompassing cognitive engagement, emotional engagement, and behavioral engagement (Kahn, 1990). Research indicates that job demands are generally negatively correlated with job engagement, while job resources are positively correlated (Bakker et al., 2007; Trépanier et al., 2014). Specifically, high job demands often increase stress, thereby inhibiting employees’ cognitive and emotional engagement, whereas sufficient job resources effectively enhance these forms of engagement (Bakker et al., 2007; Vander Elst et al., 2016). Regarding the internal mechanisms of job engagement, studies have found significant positive relationships among cognitive engagement, emotional engagement, and behavioral engagement (Yang et al., 2021; Joshi et al., 2022). Based on these theoretical and empirical findings, this study constructs a second-order chain mediation structural model to explore the mechanisms by which job demands and job resources affect gig workers’ Job engagement. The specific hypotheses are as follows:

*H3*: Job demands have a significant negative impact on gig workers' cognitive engagement.

*H4*: Job demands have a significant negative impact on gig workers' emotional engagement.

*H5*: Job resources have a significant positive impact on gig workers' cognitive engagement.

*H6*: Job resources have a significant positive impact on gig workers' emotional engagement.

*H7*: Gig workers' cognitive engagement has a significant positive impact on behavioral engagement.

*H8*: Gig workers' emotional engagement has a significant positive impact on behavioral engagement.

#### 2.3.3 Job engagement and counterproductive work behavior

Counterproductive work behavior typically refers to actions in the work environment that negatively affect productivity, efficiency, and teamwork (Balducci et al., 2011). Existing research indicates that job engagement positively influences individual work performance, organizational commitment, and organizational citizenship behavior, while negatively impacting counterproductive work behavior (Bakke, 2004; Mohsin et al., 2022). Zhang et al. (2023) note that digital gig platforms use data and algorithms to enhance management efficiency and achieve organizational goals. However, the decision-making mechanisms of algorithmic management are opaque and continuously evolving. Many gig workers report that continuous monitoring, lack of transparency, and inhumanity associated with algorithmic management create a sense of unfairness, diminish trust and satisfaction, and ultimately affect their job engagement, leading to disruptive counterproductive work behavior. Research by Sivarajan et al. (2021) and Zhao et al. (2007) also reveals that algorithmic management results in information asymmetry, leading gig workers to feel that employers have breached psychological contracts and engaged in violations. This not only directly impacts gig workers’ cognitive, emotional, and behavioral engagement but also exacerbates negative changes in these areas, further fueling counterproductive work behavior. These studies highlight the crucial role of gig workers’ job engagement in influencing counterproductive work behavior. Therefore, based on the above research, this study further proposes the following hypotheses:

*H9*: Gig workers' cognitive engagement has a significant negative impact on counterproductive work behavior.

*H10*: Gig workers' emotional engagement has a significant negative impact on counterproductive work behavior.

*H11*: Gig workers' behavioral engagement has a significant negative impact on counterproductive work behavior.

#### 2.3.4 Mediating effect of job engagement

Job engagement plays a complex mediating role between the job demands and resources on the platform and gig workers’ counterproductive work behavior. Existing research indicates that job demands necessitate sustained physical and psychological effort from workers, which can lead to burnout and negative emotions, thereby adversely affecting job engagement. Conversely, job resources provide support and assistance, help achieve work goals, alleviate work stress, and offer learning and opportunities for professional development, resulting in increased job engagement (Bakker et al., 2007; Balducci et al., 2011; Trépanier et al., 2014). Furthermore, research has shown that when individuals exhibit high levels of cognitive and emotional engagement at work, they typically display positive attitudes and emotional states. This positive mindset and emotional engagement can mitigate negative emotions and, consequently, reduce the likelihood of engaging in counterproductive work behavior (Chen et al., 2020; Sivarajan et al., 2021; Zhang et al., 2023). Additionally, existing studies suggest that job engagement encompasses cognitive, emotional, and behavioral engagement dimensions, with cognitive and emotional engagement positively impacting behavioral engagement. High levels of cognitive engagement increase the likelihood of proactive behavior, problem-solving efforts, and performance enhancement, while positive emotional experiences contribute to greater behavioral engagement and commitment (Dubovi and Tabak, 2021; Joshi et al., 2022; Yang et al., 2021). Therefore, this study proposes the following hypothesis:

*H3-1*: Job demands positively impact counterproductive work behavior among gig workers through cognitive engagement.

*H3-2*: Job demands negatively influence behavioral engagement through cognitive engagement, subsequently positively impacting counterproductive work behavior among gig workers.

*H4-1*: Job demands positively impact counterproductive work behavior among gig workers through emotional engagement.

*H4-2*: Job demands negatively influence behavioral engagement through emotional engagement, subsequently positively impacting counterproductive work behavior among gig workers.

*H5-1*: Job resources negatively impact counterproductive work behavior among gig workers through cognitive engagement.

*H5-2*: Job resources positively influence behavioral engagement through cognitive engagement, subsequently negatively impacting counterproductive work behavior among gig workers.

*H6-1*: Job resources negatively impact counterproductive work behavior among gig workers through emotional engagement.

*H6-2*: Job resources positively influence behavioral engagement through emotional engagement, subsequent negatively impacting counterproductive work behavior among gig workers.

#### 2.3.5 Moderating effects of platform formalization

Platform formalization in digital gig platforms supports collaboration through contracts, standards, processes, and structures to achieve shared goals between the platform and gig workers (Schminke et al., 2002; Chen et al., 2023). This formalization provides gig workers with a clear work framework, ensuring equitable allocation and use of authority, thereby enhancing work efficiency and quality (Howcroft and Bergvall-Kåreborn, 2019; Gol et al., 2019). Additionally, formalization is regarded as a mechanism for controlling deviant behavior (Dischner, 2015). Zhang et al. (2023) indicate that while digital gig platforms enhance management efficiency through data and algorithms, the lack of transparency and constant changes in algorithmic decisions lead to perceptions of unfairness among gig workers, reducing their trust and satisfaction, which in turn affects their job engagement and may trigger counterproductive behavior. Chen et al. (2023) find that the current governance models of gig platforms benefit the platform and customers but place gig workers in a vulnerable position. Therefore, it is essential to balance the interests of the platform, gig workers, and customers, properly regulate the current governance models, improve the work environment for gig workers, and thus enhance job engagement while reducing counterproductive work behavior. Dischner (2015) also reports a negative correlation between organizational formalization, standardization, and counterproductive work behavior. Based on these findings, this study further proposes the following hypotheses:

*H12*: Platform formalization moderates the relationship between cognitive engagement and counterproductive work behavior, weakening the negative impact of gig workers' cognitive engagement on counterproductive work behavior

*H13*: Platform formalization moderates the relationship between emotional engagement and counterproductive work behavior, weakening the negative impact of gig workers' emotional engagement on counterproductive work behavior.

*H14*: Platform formalization moderates the relationship between behavioral engagement and counterproductive work behavior, weakening the negative impact of gig workers' behavioral engagement on counterproductive work behavior.

## 3 Research methodology

This study employs a second-order structural equation model to validate a comprehensive theoretical model of gig workers’ counterproductive work behavior, based on the JD-R model and JE theory. As an advanced statistical tool, the second-order structural equation model is utilized to model and analyze multidimensional concepts and complex relationships (Koufteros et al., 2009). Compared to a first-order structural equation model, the second-order model comprehensively captures the substructure of multidimensional relationships by introducing second-order factors, providing a more nuanced understanding of the relationships between variables. The theoretical model of this study encompasses dimensions such as job demands, job resources, job engagement, and counterproductive work behavior. The second-order structural equation model is better suited to address such complex relationships. Second-order factors for job demands include work pace/workload, physical demands, psychological demands, and customer-related social stressors. Job resources include compensation, job security, learning opportunities, and opportunities for professional development. This division aids in a more detailed understanding of the impact of each dimension on gig workers’ counterproductive work behavior, making the theoretical framework more specific and operational. Furthermore, the second-order structural equation model enhances the scientific rigor and credibility of this study by assessing the fit between the model and actual data.

### 3.1 Questionnaire design and variable measurement

The survey questionnaire in this study consists of two modules: demographic information and main content. The demographic module covers information such as gender, age, occupational field, work experience, and average monthly income. The main content module includes four parts: independent variables (work pace/workload, physical demands, psychological demands, customer-related social stressors, compensation, job security, learning opportunities, and opportunities for professional development), mediating variables (cognitive, emotional, and behavioral engagement), moderating variable (platform formalization), and dependent variable (counterproductive work behavior). All constructs were measured using a 7-point Likert scale, ranging from 1 (Strongly Disagree) to 7 (Strongly Agree), with participants rating each question. The 7-point Likert scale is widely used in social science research due to its multiple advantages. Firstly, the 7-point Likert scale offers high resolution and sensitivity, capturing workers’ psychological states and behavioral tendencies in greater detail, thus enhancing the accuracy and reliability of the data (Joshi et al., 2022). Secondly, the 7-point scale provides more response options, facilitating statistical analysis and allowing researchers to explore potential patterns and relationships within the data more flexibly (Taherdoost, 2019). In summary, the application of the 7-point Likert scale in this study not only improves the precision of data collection but also enhances the potential for data analysis and the interpretability of research findings.

To enhance the scale’s adaptability, we implemented the following measures: Firstly, the scale was translated from English to Chinese by professional translators and underwent multiple rounds of proofreading to ensure semantic accuracy. Secondly, a pre-survey involving 216 participants was conducted to validate the reliability and validity of the translated questionnaire, with necessary revisions made based on feedback. All formal surveys were administered in Chinese to ensure respondents could accurately comprehend the questions and provide genuine and reliable feedback. These steps significantly improved the scale’s adaptability and ensured the accuracy and reliability of the survey results (Ho, 2024). For job demands, work pace/workload, physical demands, and psychological demands are measured using scales adapted from Bakker et al. (2003), tailored to the characteristics of gig platforms. Example questions include: “Do you work under time constraints?” “In your job, do you feel fatigued or uncomfortable due to prolonged, repetitive movements?” and “Do you need to stay focused on your work continuously?.” Customer-related social stressors are measured using the scale by Dormann and Zapf (2004), with questions like: “Customers vent their frustrations on us.” Each variable is measured with five items. The measurement of job resources, including compensation, learning opportunities, and opportunities for professional development, refers to the scale by Bakker et al. (2003) with appropriate modifications. Example questions include: “Do you feel your salary is sufficient for a comfortable life?” “Does your work allow you to think and act independently?” and “Does your company provide training opportunities?” Job security is measured using the scale by Kraimer et al. (2005), with questions like: “The company will not reduce my weekly working hours.” Each variable in job resources is measured with five items, except for opportunities for professional development, which is measured with four items. Cognitive, emotional, and behavioral engagement of gig workers are measured using a well-established scale by Rich et al. (2010), modified appropriately for gig platform characteristics. Example questions include: "During work, I am highly attentive to my tasks.” “I feel energetic at work.” and “I work very hard.” Each variable is measured with five items. Counterproductive work behavior is assessed using the refined scale developed by Koopmans et al. (2014), comprising the following five items: “I exaggerate problems at work.” “I complain about unimportant things at work.” “I focus on the negative aspects of work rather than the positive.” “I discuss the negative aspects of my work with others.” and “Sometimes I should be working, but I do nothing.” Platform formalization is measured based on the scale presented by Schminke et al. (2002) and has been appropriately modified to align with the characteristics of gig platforms. The assessment includes the following five inquiries: “The company adopts numerous written regulations and policies to efficiently manage work processes.” “There is an easily accessible “Rules and Procedures” manual providing guidance for gig workers.” “The majority of positions have comprehensive written job descriptions, specifying responsibilities and tasks.” “The company records the work performance of each gig worker to ensure transparency and fairness in evaluations” and “Most gig workers undergo onboarding training.” A detailed questionnaire is provided in Appendix Table A1.

### 3.2 Data collection and common method bias test

Shanghai, a global leader in the digital economy, has fostered the rapid development of digital gig platforms through its advanced information technology and extensive networking capabilities, creating a gig market of approximately 3 million workers. This provides a rich source of sample data for studying counterproductive work behavior among gig workers on digital platforms (Shanghai Municipal Human Resources and Social Security Bureau, 2023a,b). Furthermore, the Shanghai municipal government’s “18 Measures” employment stabilization policy underscores the significance of gig market development, offering robust policy support through the establishment of dedicated gig zones on public platforms, enhanced recruitment services, and encouragement of social capital and human resource agencies’ involvement (Shanghai Municipal Human Resources and Social Security Bureau, 2023a,b). Finally, as a leading city in the global digital economy, Shanghai’s gig market development is of notable international influence and representativeness, providing valuable insights for the evolution of gig markets within the global digital economy context (Cui and Shi, 2012). Consequently, this study utilized the professional survey platform “sojump” to conduct a questionnaire survey among gig workers on digital platforms in Shanghai, China. To ensure the accuracy of the questionnaire sample, two screening questions were designed. The first question inquired, “Are you a gig worker? (Yes/No),” while the second questioned, “What type of gig work do you engage in? (Online gig work, offline gig work, or both).” Only respondents answering “Yes” and selecting “Online gig work or both” and having an IP address located in Shanghai were included in the sample. The survey was conducted in December 2023. Before the official online survey, we conducted a preliminary test with 216 questionnaires, predicting an average response time of approximately 8 min. To ensure data validity and exclude inattentive responses, we set completion times below 200 s or above 1,000 s as invalid (Su et al., 2023). In total, 856 valid questionnaires were collected, with an effectiveness rate of 70.337%. For further details, refer to Table 1. This study utilized the Harman single-factor analysis to conduct factor analysis on all items, with the total variance explained by the first principal component being 21.257%. Preliminary results indicate the absence of common method bias in the data.

**Table 1:** Respondent’s profile.

Characteristics	Items	Frequency (*n* = 856)	Percentage (%)
Gender	Male	443	51.8
	Female	413	48.2
Age (years)	0–20	38	4.4
	21–30	278	32.5
	31–40	295	34.5
	41–50	193	22.5
	51–60	29	3.4
	Above 60	23	2.7
Occupational fields	Transportation, storage, and postal services	222	25.9
	Accommodation and catering services	206	24.1
	Real estate industry	150	17.5
	Scientific research and technical services industry	59	6.9
	Information transmission, computer services, and software	169	19.7
	Other	50	5.8
Work experience (years)	Less than 1	77	9.0
	1–3	274	32.0
	4–10	348	40.7
	More than 10	157	18.3
Average monthly income (RMB)	0–1,000	119	13.9
	1,001–4,000	331	38.7
	4,001–7,000	241	28.2
	7,001–10,000	146	17.1
	More than 10,000	19	2.2

## 4 Research findings

### 4.1 Confirmatory factor analysis

This study conducted a confirmatory factor analysis to assess the structural validity among variables, and specific results are presented in Table 2. The Cronbach’s alpha coefficients and composite reliability (CR) values for each variable exceeded 0.7. Additionally, standardized loading coefficients and the average variance extracted (AVE) values surpassed 0.5. These outcomes indicate robust results in terms of reliability and validity analysis for the study questionnaire (Hair et al., 2019). Furthermore, the study employed Variance Inflation Factor (VIF) to check for collinearity among constitutive measurement items. The VIF values for all measurement items ranged from 1.131 to 2.277, all below 3, suggesting the absence of significant collinearity issues in the data (Hair et al., 2019). Consequently, these data are deemed suitable for subsequent structural model analysis.

**Table 2:** Results of confirmatory factor analysis.

Construct	Item	Cronbach’s alpha	Factor loading	Average variance extracted	Composite reliability
Counterproductive work behavior	CWB1	0.893	0.788	0.626	0.893
	CWB2		0.826		
	CWB3		0.775		
	CWB4		0.789		
	CWB5		0.776		
Cognitive engagement	CE1	0.884	0.744	0.606	0.885
	CE2		0.810		
	CE3		0.790		
	CE4		0.751		
	CE5		0.794		
Emotional engagement	EE1	0.915	0.848	0.685	0.916
	EE2		0.831		
	EE3		0.839		
	EE4		0.815		
	EE5		0.803		
Behavioral engagement	BE1	0.898	0.788	0.638	0.898
	BE2		0.825		
	BE3		0.798		
	BE4		0.800		
	BE5		0.782		
Work pace/workload	WPW1	0.877	0.793	0.590	0.878
	WPW2		0.749		
	WPW3		0.802		
	WPW4		0.793		
	WPW5		0.698		
Physical demands	PHD1	0.883	0.762	0.602	0.883
	PHD2		0.770		
	PHD3		0.767		
	PHD4		0.796		
	PHD5		0.785		
Psychological demands	PSD1	0.890	0.802	0.620	0.891
	PSD2		0.791		
	PSD3		0.748		
	PSD4		0.806		
	PSD5		0.787		
Customer-related social stressors	CSS1	0.867	0.760	0.568	0.868
	CRSS2		0.736		
	CRSS3		0.799		
	CRSS4		0.754		
	CRSS5		0.718		
Compensation	CO1	0.896	0.802	0.632	0.896
	CO2		0.792		
	CO3		0.802		
	CO4		0.802		
	CO5		0.775		
Job security	JS1	0.860	0.770	0.553	0.861
	JS2		0.743		
	JS3		0.743		
	JS4		0.763		
	JS5		0.696		
Learning opportunities	LO1	0.895	0.810	0.631	0.895
	LO2		0.806		
	LO3		0.787		
	LO4		0.799		
	LO5		0.768		
Opportunities for professional development	OPD1	0.836	0.723	0.561	0.836
	OPD2		0.780		
	OPD3		0.727		
	OPD4		0.765		
Platform formalization	PF1	0.898	0.744	0.606	0.885
	PF2		0.810		
	PF3		0.790		
	PF4		0.751		
	PF5		0.794		

Model fit indices: χ^2^/df = 1.741 (*p* < 0.001); NFI = 0.910; RFI = 0.906; RMSEA = 0.029; SRMR = 0.0553.

### 4.2 Correlation analysis

The results of the correlation analysis among variables are presented in Table 3. Firstly, there is a significant correlation among the four first-order variables of job demands (Work Pace/Workload, Physical Demands, Psychological Demands, Customer-related social stressors) and job resources (Compensation, Job Security, Learning Opportunities, Opportunities for professional development). Secondly, there is a significant positive correlation between cognitive, emotional engagement, and behavioral engagement. Finally, there is a significant negative correlation between cognitive, emotional, and behavioral engagement, and counterproductive work behavior. Moreover, all correlation coefficients are below 0.75, and the square root of AVE is greater than the correlation coefficients. These preliminary results of the correlation analysis validate the relationship assumptions, providing a robust foundation for subsequent research.

**Table 3:** Average variance extracted and squared correlations of the constructs.

	Counterproductive work behavior	Cognitive engagement	Emotional engagement	Behavioral engagement	Work pace/workload	Physical demands	Psychological demands	Customer-related social stressors	Compensation	Job security	Learning opportunities	Opportunities for professional development	Platform formalization
Counterproductive work behavior	0.791												
Cognitive engagement	−0.431**	0.778											
Emotional engagement	−0.352**	0.356**	0.828										
Behavioral engagement	−0.422**	0.411**	0.353**	0.799									
Work pace/workload	0.134**	−0.134**	−0.138**	−0.097**	0.768								
Physical demands	0.215**	−0.225**	−0.220**	−0.214**	0.500**	0.776							
Psychological demands	0.034	0.047	−0.009	0.069*	0.469**	0.328**	0.787						
Customer-related social stressors	0.137**	−0.101**	−0.128**	−0.078*	0.499**	0.404**	0.524**	0.754					
Compensation	−0.199**	0.258**	0.285**	0.251**	−0.081*	−0.137**	−0.069*	−0.105**	0.795				
Job security	−0.238**	0.300**	0.312**	0.252**	−0.122**	−0.146**	−0.084*	−0.129**	0.573**	0.744			
Learning opportunities	−0.294**	0.446**	0.400**	0.370**	−0.063	−0.193**	0.087*	−0.063	0.565**	0.552**	0.794		
Opportunities for professional development	−0.232**	0.347**	0.396**	0.307**	−0.080*	−0.168**	0.007	−0.081*	0.622**	0.511**	0.643**	0.749	
Platform formalization	−0.147**	0.200**	0.221**	0.229**	0.034	−0.071*	0.146**	0.049	0.078*	0.131**	0.208**	0.202**	0.778

** Significant at the 0.01 level (two-tailed), indicating significant correlation.*Significant at the 0.05 level (two-tailed), indicating significant correlation. Average variance extracted are along the main diagonal. Squared correlations between constructs are above the main diagonal.

### 4.3 Structural model examination

**Figure 2:**
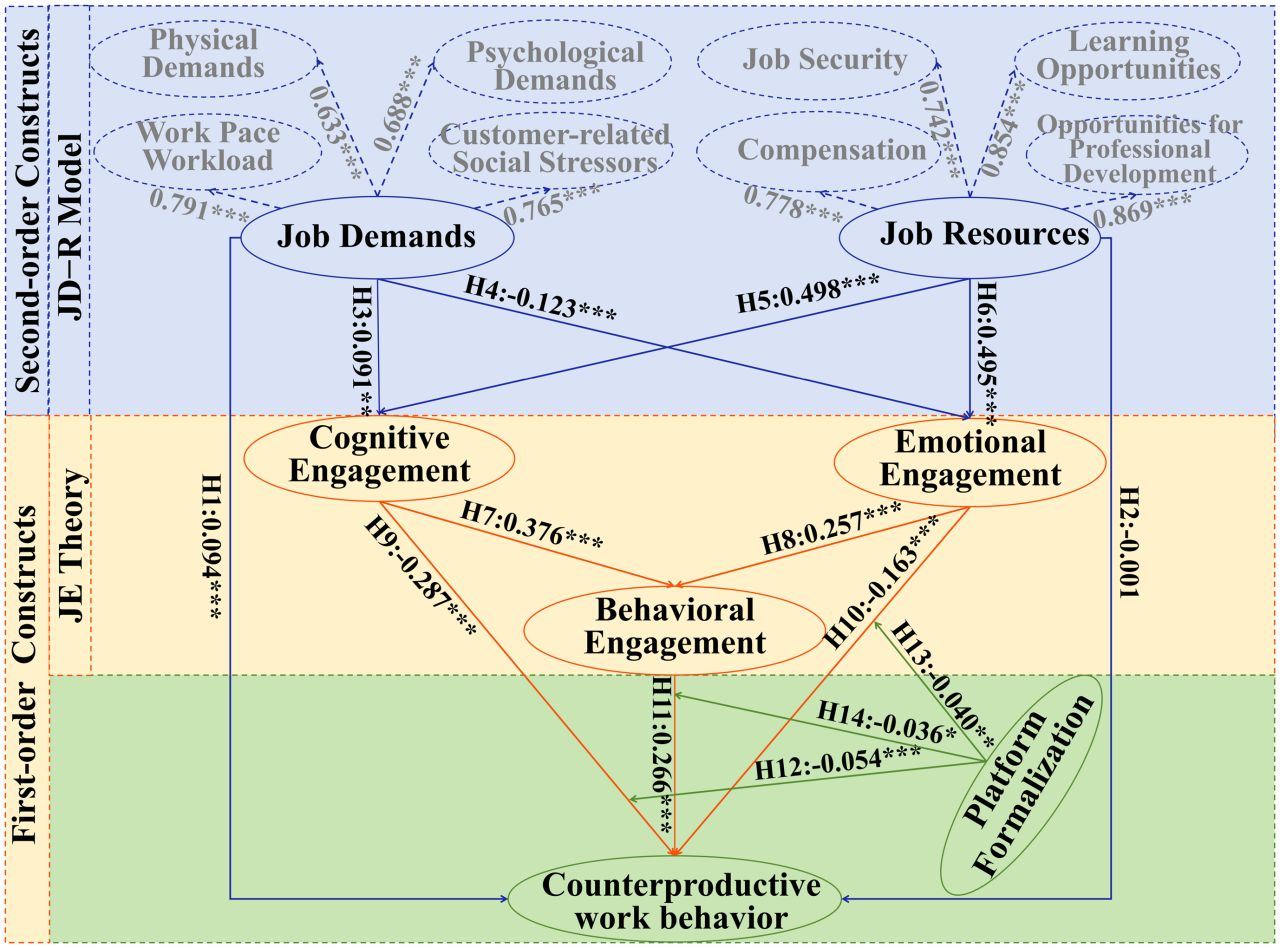
Structural modeling result.

To delve into the relationships among variables, this study, grounded in the JD-R model and JE theory, constructed a a second-order chain mediation structural model using Amos 26.0. The comprehensive theoretical model was subjected to scrutiny through structural equation modeling. The results of the path analysis are depicted in Figure 2 and Table 4. The overall model fit indices are Chi-Square Statistic to Degrees of Freedom Ratio (*χ^2^/df*) = 1.769 (*p* < 0.001), NFI = 0.909, RFI = 0.904, RMSEA = 0.030, SRMR = 0.066, indicating a good fit between the data and the model. The *p*-values for H1 and H3-11 are all below 0.05, supporting the validity of these hypotheses. However, the p-value for H2 exceeds 0.05, thus the hypothesis is not supported. Comparing the absolute values of standardized path coefficients reveals that, first, job demands have a significant positive direct effect on counterproductive work behavior, while job resources do not have a significant effect. Second, job demands have a significant negative effect on both cognitive and emotional engagement, whereas job resources have the opposite effect. Third, cognitive and emotional engagement have a significant positive effect on behavioral engagement. Lastly, cognitive, emotional, and behavioral engagement all have significant negative effects on counterproductive work behavior.

**Table 4:** Results of hypothesis testing.

Hypothesis	Path	Coefficients	*P*-value	Test results
H1	Job demands → Counterproductive work behavior	0.094	***	Supported
H2	Job resources → Counterproductive work behavior	−0.001	0.977	Not Supported
H3	Job demands → Cognitive engagement	−0.091	**	Supported
H4	Job demands → Emotional engagement	−0.123	***	Supported
H5	Job resources → Cognitive engagement	0.498	***	Supported
H6	Job resources → Emotional engagement	0.495	***	Supported
H7	Cognitive engagement → Behavioral engagement	0.376	***	Supported
H8	Emotional engagement → Behavioral engagement	0.257	***	Supported
H9	Cognitive engagement → Counterproductive work behavior	−0.287	***	Supported
H10	Emotional engagement → Counterproductive work behavior	−0.163	***	Supported
H11	Behavioral engagement → Counterproductive work behavior	−0.266	***	Supported

*** Significant at the 0.01 level (two-tailed), indicating significant correlation. ** Significant at the 0.05 level (two-tailed), indicating significant correlation. Structural model fit indices: χ^2^/df = 1.769 (*p* < 0.001), NFI = 0.909, RFI = 0.904, RMSEA = 0.030, SRMR = 0.066.

### 4.4 Mediation analysis

To further investigate the mediating effects of cognitive, emotional, and behavioral engagement on counterproductive work behavior, this study employed Amos 26.0 software for 5,000 bootstrap analyses, establishing a 95% confidence interval to examine the mediating effects of intermediate variables. The analysis results are presented in Table 5. Firstly, Job demands significantly positively influence counterproductive work behavior through cognitive and emotional engagement. Secondly, Job demands significantly negatively affect behavioral engagement through cognitive and emotional engagement, which subsequently leads to a significant positive impact on counterproductive work behavior. Thirdly, Job resources significantly negatively influence counterproductive work behavior through cognitive and emotional engagement. Finally, Job resources significantly positively affect behavioral engagement through cognitive and emotional engagement, which subsequently leads to a significant negative impact on counterproductive work behavior.

**Table 5:** Mediation effect analysis results.

Hypothesis	Path	Estimate	Bias-corrected 95% CI		*P*-value	Test results
			Lower	Upper		
H3-1	Job demands → Cognitive engagement → Counterproductive work behavior	0.037	0.007	0.075	***	Supported
H4-1	Job demands → Emotional engagement → Counterproductive work behavior	0.028	0.010	0.058	***	Supported
H3-2	Job demands → Cognitive engagement → Behavioral engagement → Counterproductive work behavior	0.013	0.003	0.027	***	Supported
H4-2	Job demands → Emotional engagement → Behavioral engagement → Counterproductive work behavior	0.012	0.004	0.023	***	Supported
Total indirect effect		0.089	0.036	0.150	***	Supported
Direct effect		0.131	0.027	0.241	***	Supported
Total effect		0.221	0.113	0.340	***	Supported
H5-1	Job resources → Cognitive engagement → Counterproductive work behavior	−0.198	−0.279	−0.133	***	Supported
H6-1	Job resources → Emotional engagement → Counterproductive work behavior	−0.112	−0.181	−0.054	***	Supported
H5-2	Job resources → Cognitive engagement → Behavioral engagement → Counterproductive work behavior	−0.069	−0.104	−0.044	***	Supported
H6-2	Job resources → Emotional engagement → Behavioral engagement → Counterproductive work behavior	−0.047	−0.074	−0.028	***	Supported
Total indirect effect		−0.425	−0.536	−0.330	***	Supported
Direct effect		−0.002	−0.131	0.127	0.976	Not supported
Total effect		−0.427	−0.546	−0.317	***	Supported

*** Significant at the 0.01 level (two-tailed), indicating significant correlation. ** Significant at the 0.05 level (two-tailed), indicating significant correlation.

### 4.5 Moderation effects examination

**Figure 3:**
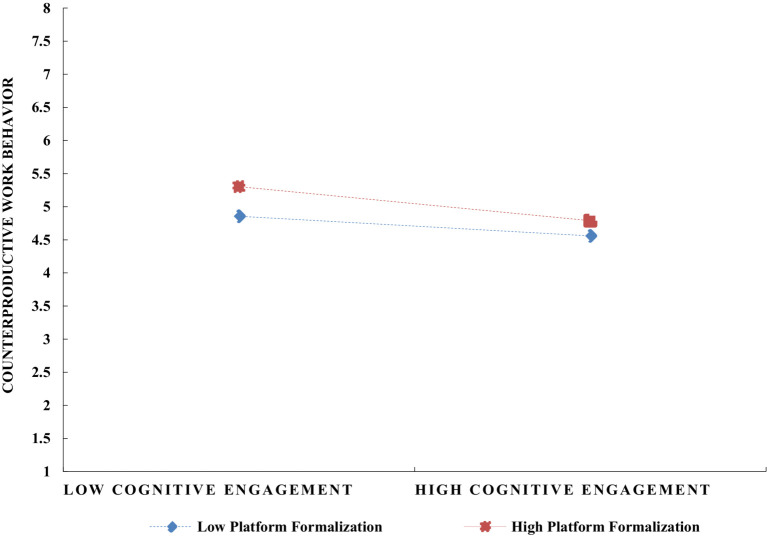
The moderating effect of platform formalization on the relationship between gig workers’ cognitive engagement and counterproductive work behavior.

**Figure 4 fig4:**
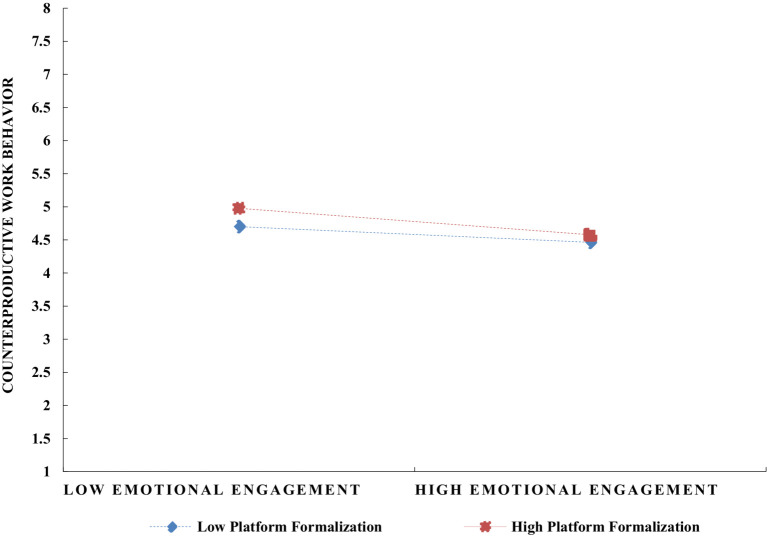
The moderating effect of platform formalization on the relationship between gig workers’ emotional engagement and counterproductive work behavior.

**Figure 5:**
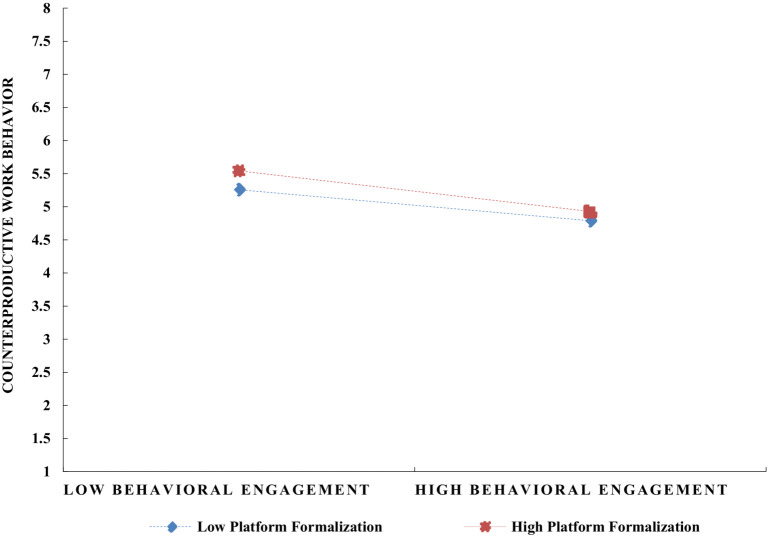
The moderating effect of platform formalization on the relationship between gig workers’ behavioral engagement and counterproductive work behavior.

To explore the moderating role of platform formalization on the relationship between job engagement and counterproductive work behavior in digital gig platforms, this study utilized SPSS 27 for the corresponding moderation effect analysis, with results presented in Table 6. To clearly illustrate the moderating effect of platform formalization, the study classified it into high and low levels by adding and subtracting one standard deviation, and depicted the interaction effects in Figures 3 [Fig fig4]–5. The simple slope analysis reveals that platform formalization moderates the relationship between cognitive, emotional, and behavioral engagement and counterproductive work behavior. Specifically, platform formalization mitigates the negative impact of cognitive, emotional, and behavioral engagement on counterproductive work behavior, thus providing further support for Hypotheses 12–14.

**Table 6:** Moderation effect analysis results.

Hypothesis	Independent variable	Coeff	SE	*T*	*P*-value	LLCI	ULCI
H12	Constant	4.8770	0.4000	12.1929	***	4.0919	5.6621
	Cognitive engagement	−0.2029	0.0930	−2.1818	**	−0.3854	−0.0204
	Platform formalization	0.1694	0.0875	1.9360	*	−0.0023	0.3412
	Platform formalization* Cognitive engagement	−0.0544	0.0196	−2.7788	***	−0.0928	−0.0160
H13	Constant	4.679	0.360	12.986	***	3.972	5.387
	Emotional engagement	−0.159	0.085	−1.879	*	−0.326	0.007
	Platform formalization	0.098	0.082	1.188	0.235	−0.064	0.260
	Platform formalization* Emotional engagement	−0.040	0.019	−2.175	**	−0.077	−0.004
H14	Constant	5.1293	0.3997	12.8342	***	4.3449	5.9138
	Behavioral engagement	−0.2704	0.0912	−2.9638	***	−0.4494	−0.0913
	Platform formalization	0.1062	0.0891	1.1920	0.234	−0.0687	0.2810
	Platform formalization* Behavioral engagement	−0.0361	0.0194	−1.8607	*	−0.0742	0.0020

*** Significant at the 0.01 level (two-tailed), indicating significant correlation. ** Significant at the 0.05 level (two-tailed), indicating significant correlation. * Significant at the 0.1 level (two-tailed), indicating significant correlation.

### 4.6 Discussion of research findings

This study developed a second-order chain mediation structural model to investigate how job demands and resources on digital gig platforms influence gig workers’ counterproductive work behavior through job engagement. The following is a detailed analysis and discussion of the research findings: First, job demands have a significant positive impact on gig workers’ counterproductive work behavior. Further analysis reveals that job demands also indirectly influence counterproductive work behavior through cognitive and emotional engagement as mediators. This phenomenon may be associated with the use of algorithmic technologies for task allocation and performance evaluation on digital gig platforms. Specifically, the lack of transparency and humanization in algorithmic management creates information asymmetry between the platform and gig workers, weakening workers’ perception of the platform and potentially diminishing their cognitive engagement (Chen et al., 2023; Zhang et al., 2023). Additionally, high work pace and customer-related social stressors may induce negative emotions such as anxiety, stress, and frustration among gig workers, which can lower their emotional engagement and increase the likelihood of counterproductive work behavior (Tan et al., 2021; Zhang et al., 2023). In contrast to job demands, the results indicate that job resources reduce counterproductive work behavior by influencing cognitive, emotional, and behavioral engagement. This suggests that providing gig workers with adequate compensation, job security, learning opportunities, and opportunities for professional development can encourage greater job engagement, thereby helping to reduce their counterproductive work behavior (Duggan et al., 2022).

Secondly, job demands within the digital gig platform negatively influence behavioral engagement through cognitive and emotional engagement, subsequently positively impacting counterproductive work behavior. This indicates that excessive job demands, such as an overwhelming workload and job pressure, may lead to a decrease in gig workers’ cognitive and emotional engagement, affecting their behavioral engagement (Yang et al., 2021; Joshi et al., 2022), and increasing the likelihood of exhibiting counterproductive work behavior (Sivarajan et al., 2021; Zhao et al., 2007). This finding further supports the positive impact mechanism of job demands on counterproductive work behavior, emphasizing the mediating role of cognitive, emotional, and behavioral engagement. Conversely, job resources positively influence behavioral engagement through cognitive and emotional engagement, subsequently negatively impacting counterproductive work behavior. In other words, favorable job resources may encourage gig workers to be more actively engaged in their work, increasing cognitive and emotional engagement while decreasing the likelihood of exhibiting counterproductive work behavior (Granger et al., 2022). This underscores the promoting role of job resources in fostering gig workers’ active participation in work and reducing counterproductive work behavior. Through an in-depth analysis of the chain-mediated effects, the study reveals the mechanisms through which job demands and job resources influence gig workers’ behavioral engagement and counterproductive work behavior, confirming the significant positive impact of cognitive and emotional engagement on behavioral engagement.

Finally, the research unveils that the formalization of digital gig platforms plays a moderating role in the relationship between cognitive, emotional, and behavioral engagement and counterproductive work behavior. It diminishes the negative impact of gig workers’ job engagement on counterproductive work behavior. Specifically, the standardized platform environment provides explicit work guidelines and expectations, enhancing gig workers’ cognitive awareness. Simultaneously, governance through formalization reduces workers’ sense of unfairness, encouraging more proactive engagement with work (Chen et al., 2023). This regulatory governance diminishes the adverse effects of cognitive, emotional, and behavioral engagement on counterproductive work behavior, motivating workers to fulfill their responsibilities more effectively. Consequently, it enables effective performance monitoring of gig workers and reduces the occurrence of counterproductive work behavior (Howcroft and Bergvall-Kåreborn, 2019).

## 5 Research conclusions

### 5.1 Theoretical contributions

This study contributes to the understanding of the complex relationship between job demands-resources and counterproductive work behavior on digital gig platforms in three main ways. Firstly, the JD-R model has predominantly been applied in traditional work settings, where definitions and elements of job demands and resources are relatively clear. However, the decentralized and flexible nature of digital gig platforms necessitates a re-evaluation of these definitions (Koufteros et al., 2009). By conceptualizing job demands and resources as second-order factors and considering multiple dimensions, we extend the applicability of the JD-R model to digital gig platforms. This multidimensional approach enhances our understanding of the work environment on digital gig platforms and provides a more comprehensive and precise theoretical framework for understanding and predicting counterproductive work behavior, addressing gaps in current research regarding the impact of the work environment on counterproductive behavior among gig workers (Bakker et al., 2007; Watson et al., 2021).

Secondly, this study applies the JE theory to explore how job demands and resources on digital gig platforms affect counterproductive work behavior through job engagement. This theory disaggregates job engagement into cognitive, emotional, and behavioral dimensions, effectively addressing the limitations of previous research that used the work engagement but overlooked the overall work environment and organizational engagement aspects (Shuck et al., 2017). This approach also provides a more nuanced and detailed analysis of gig workers’ job attitudes and counterproductive work behavior. Additionally, applying the JE theory to the study of counterproductive work behavior among gig workers expands the theoretical framework’s scope.

Lastly, although existing research has explored the necessity of formalization in digital gig platforms (Chen et al., 2023), there has been limited analysis of its impact on the relationship between job engagement and counterproductive behavior. This study fills this gap, offering new insights into how platform formalization affects gig workers’ behavior and highlighting its importance in optimizing the work environment, enhancing job engagement, and reducing counterproductive behavior. These findings not only provide new theoretical support for understanding gig worker behavior on digital platforms but also offer important implications for future organizational management and behavioral research.

### 5.2 Practical implications

The empirical findings of this study offer practical insights for managers of digital gig platforms, aiding in the development of effective strategies to reduce gig workers’ counterproductive work behavior. Specific practical recommendations are as follows: Firstly, designing job demands appropriately. Platform managers should leverage data analytics to gain an in-depth understanding of gig workers’ abilities and personal preferences. Based on this understanding, continuously optimize task allocation algorithms to ensure that the tasks assigned take into account gig workers’ physical conditions and capacity limits (Xiongtao et al., 2021). Tasks’ difficulty, quantity, and deadlines should be kept within a reasonable range to avoid inducing stress and fatigue due to excessive workload or rapid work pace, thereby reducing the occurrence of counterproductive work behavior among gig workers.

Secondly, enhance gig workers’ job engagement. Managers should first focus on improving cognitive engagement among gig workers by providing regular training to elevate their knowledge, skills, and service capabilities. For instance, organizing online or offline skill development workshops and career advancement seminars can increase cognitive engagement, better equipping workers to meet platform and customer demands (Nilsen and Kongsvik, 2023). Next, bolster emotional engagement by establishing effective communication channels and timely feedback mechanisms to ensure smooth information flow. For example, setting up online customer service to offer practical and emotional support, and creating support groups to provide immediate assistance and problem-solving solutions can alleviate customer-related social stressors and work-related stress (Balducci et al., 2011; Zhao et al., 2007). To stimulate behavioral engagement, design diverse and reasonable incentive and reward mechanisms for gig workers. This could include compensation rewards, recognition, and opportunities for promotion, as well as implementing monthly or quarterly awards for outstanding workers to boost their enthusiasm and job engagement (Bakker et al., 2007). Additionally, focus on the work environment by fostering a positive and fair atmosphere, such as hosting regular online and offline team-building activities to enhance team cohesion and job satisfaction (Priesemuth et al., 2013).

Lastly, platform formalization governance. Managers should standardize platform operations by establishing clear work guidelines and expectations. This includes publishing detailed workflow guides and conduct manuals to ensure gig workers are aware of the platform’s standards and requirements (Chen et al., 2023). By organizing training sessions to explain the basic principles and operational mechanisms of algorithmic management, managers can enhance the transparency of performance assessments and reward-punishment mechanisms, effectively reducing resistance and fostering a harmonious work environment (Zhang et al., 2023). Finally, provide ongoing feedback and evaluation mechanisms, such as regular work assessments and performance review meetings, to help gig workers understand their performance and receive improvement suggestions.

### 5.3 Research limitations and future directions

Although this study has made significant contributions to understanding the relationships between job demands, resources, and counterproductive work behavior on digital gig platforms, several limitations must be acknowledged. First, the sample used in this study predominantly comes from digital gig platforms in specific regions, which may introduce regional specificity and limit the generalizability of the findings. Future research could expand the sample to include a broader range of geographic locations to validate the external validity of the results. Second, this study primarily relies on self-reported data from gig workers, which may be subject to self-reporting bias. Future research could incorporate multiple data sources, such as customer reviews and peer evaluations, to obtain more comprehensive and objective data, thereby enhancing the credibility of the findings (Donaldson and Grant-Vallone, 2002). Finally, while the study considered the moderating role of platform formalization, it did not delve deeply into how internal governance mechanisms within platforms affect gig worker behavior. Future research could further analyze various dimensions of internal governance mechanisms (Waldkirch et al., 2021), such as rules and policies, incentive and punishment systems, supervision and auditing, communication and feedback channels, resource support, benefit distribution mechanisms, and conflict resolution processes, to gain a more comprehensive understanding of how platforms influence gig workers’ counterproductive work behavior.

## Data Availability

The original contributions presented in the study are included in the article/supplementary material, further inquiries can be directed to the corresponding author/s.

## Appendix

**Table A1:** Measurement items and source

Construct	ID	Measurement	Source
Counterproductive work behavior	CWB1	I exaggerate problems at work.	Koopmans et al. (2014)
	CWB2	I complain about unimportant things at work.	
	CWB3	I focus on the negative aspects of work rather than the positive.	
	CWB4	I discuss the negative aspects of my work with others.	
	CWB5	Sometimes I should be working, but I do nothing.	
Cognitive engagement	CE1	During work, I am highly focused on my tasks.	Rich et al. (2010)
	CE2	During work, my thoughts are concentrated on the job.	
	CE3	During work, I am highly attentive to my tasks.	
	CE4	During work, I am deeply absorbed in my tasks.	
	CE5	During work, I channel my energy into my tasks.	
Emotional engagement	EE1	I am interested in my work.	Rich et al. (2010)
	EE2	I am passionate about my work.	
	EE3	I feel energetic at work.	
	EE4	I am excited about my work.	
	EE5	I take pride in my work.	
Behavioral engagement	BE1	I work very hard.	Rich et al. (2010)
	BE2	I put my full effort into my work.	
	BE3	I make maximum efforts to complete my tasks.	
	BE4	I invest a significant amount of energy into my work.	
	BE5	I strive to excel in my work.	
Work pace and workload	WPW1	Do you have too much work to handle?	Bakker et al. (2003)
	WPW2	Do you feel the work pace is too fast?	
	WPW3	Do you work under time constraints?	
	WPW4	Do you need extra effort to complete tasks?	
	WPW5	Do you find yourself lagging behind in work?	
Physical demands	PHD1	Does your work require significant physical effort?	Bakker et al. (2003)
	PHD2	In your work, do you feel fatigued or uncomfortable due to activities like lifting, bending, or climbing?	
	PHD3	In your work, do you feel fatigued or uncomfortable due to prolonged, repetitive movements?	
	PHD4	Does your work feel physically demanding?	
	PHD5	Does your work make you feel fatigued or uncomfortable?	
	PHD6	Does your work require significant physical effort?	
Psychological demands	PSD1	Does your work require a high level of concentration?	Bakker et al. (2003)
	PSD2	Do you need to stay focused on your work continuously?	
	PSD3	Do you need to juggle many tasks simultaneously?	
	PSD4	In your work, do you need to engage in continuous thinking?	
	PSD5	In your work, do you need to remember many things?	
Customer-related social stressors	CSS1	Some customers always make special requests.	Dormann and Zapf (2004)
	CSS2	Customers vent their frustrations on us.	
	CSS3	When mistakes occur, customers usually blame us instead of themselves.	
	CSS4	When we are busy, customers may not be aware of it.	
	CSS5	Our customers often complain without valid reasons.	
Compensation	CO1	Do you think the company’s pay level is satisfactory?	Bakker et al. (2003)
	CO2	Do you feel your salary is sufficient for a comfortable life?	
	CO3	Do you think the compensation for your work is adequate?	
	CO4	Compared to others, do you consider your compensation fair?	
	CO5	Do you feel the company’s compensation is lower than similar companies?	
Job security	JS1	My job is stable.	Kraimer et al. (2005)
	JS2	As long as I want, my job will always be there.	
	JS3	The company will not reduce my weekly working hours.	
	JS4	If my company faces economic issues, I believe I will not be the first to be laid off.	
	JS5	If my job is terminated, the company will provide me with another job.	
Learning opportunities	LO1	Can you learn new things in your work?	Bakker et al. (2003)
	LO2	Does your work allow you to think and act independently?	
	LO3	Does your work provide growth opportunities?	
	LO4	Does your work offer development opportunities?	
	LO5	Does your work enable you to achieve accomplishments?	
Opportunities for professional development	OPD1	Does your work contribute to financial advancement?	Bakker et al. (2003)
	OPD2	Does your work increase your opportunities in the workplace?	
	OPD3	Does your company provide training opportunities?	
	OPD4	Does your work offer possibilities for promotion?	
Platform formalization	PF1	The company adopts numerous written regulations and policies to efficiently manage work processes.	Schminke et al. (2002)
	PF2	There is an easily accessible “Rules and Procedures” manual providing guidance for gig workers.	
	PF3	The majority of positions have comprehensive written job descriptions, specifying responsibilities and tasks.	
	PF4	The company records the work performance of each gig worker to ensure transparency and fairness in evaluations.	
	PF5	Most gig workers undergo onboarding training.	
